# Bone Metastases from Intrahepatic Cholangiocarcinoma Confer Worse Prognosis

**DOI:** 10.3390/curroncol30030199

**Published:** 2023-02-22

**Authors:** Ingrid Garajová, Fabio Gelsomino, Massimiliano Salati, Francesco Leonardi, Stefania De Lorenzo, Alessandro Granito, Francesco Tovoli

**Affiliations:** 1 Medical Oncology Unit, University Hospital of Parma, 43125 Parma, Italy; 2 Department of Oncology and Hematology, University Hospital of Modena, 41124 Modena, Italy; 3 Oncology Unit, Azienda USL Bologna, 40139 Bologna, Italy; 4 Division of Internal Medicine, Hepatobiliary and Immunoallergic Diseases, IRCCS Azienda Ospedaliero-Universitaria di Bologna, 40138 Bologna, Italy

**Keywords:** cholangiocarcinoma, intrahepatic, bone metastases, outcome, survival

## Abstract

Background: Metastatic intrahepatic cholangiocarcinoma still has a dismal prognosis. The aim of our study was to investigate the prognostic role of bone metastases in patients affected by intrahepatic cholangiocarcinoma. Methods: A total of 186 metastatic intrahepatic cholangiocarcinoma patients were retrospectively reviewed. Clinicopathologic and survival data were collected and reviewed, in particular overall survival, progression-free survival after first-line treatment and time from end of first-line therapy to cancer death. Results: Around 11% of intrahepatic cholangiocarcinoma patients developed bone metastases. This subgroup of patients showed no differences in progression-free survival to first-line chemotherapy but had a shorter median overall survival of 4 months compared to the group with liver involvement only (*p* = 0.03). If treated, the outcome for ECOG PS 2 patients with bone metastases was worse in comparison to patients with liver involvement only with poor performance status (*p* = 0.003). The presence of bone metastases, poor performance status and no subsequent second-line treatment was associated with a worse outcome in multivariate analysis. Conclusions: Patients with intrahepatic carcinoma and bone metastases with poor ECOG performance status might be treated with best supportive care and not active chemotherapy treatment, the decisions which have to be shared with patients.

## 1. Introduction

Cholangiocarcinoma (CCA) is a heterogeneous group of neoplasms of the bile ducts. CCA can be divided according to their anatomical location into intrahepatic (ICC) and extrahepatic CCA (ECC) [1,2]. ICC is the second most frequent primary liver cancer, following hepatocellular carcinoma (HCC). Compared with HCC, ICC is more invasive and has a higher probability of metastasis.

ICCs originate from the intrahepatic biliary tree and account for 10−20% of all CCA cases. The overall incidence rate of ICCs is increasing worldwide, as well as mortality due to late diagnosis, tumor aggressiveness and treatment resistance [2,3]. Incidence of cholangiocarcinomas varies throughout the world due to different distribution of risk factors. In Western countries, the most common risk factors are fibropolycystic liver disease and primary sclerosing cholangitis. In Asia, the main cause is chronic infection with liver flukes. Other risk factors include chronic liver disease, hepatolithiasis, alcohol, smoking, obesity, fatty liver disease, diabetes, inflammatory bowel disease and certain toxic exposures. Moreover, some genetic conditions are associated with cholangiocarcinoma (for example, Lynch syndrome) [1,2,3]. Regarding risk factors, choledochal cysts are strongly associated with both ICC and ECC, while cirrhosis is more strongly associated with ICC than ECC, and choledocholithiasis is more strongly associated with ECC than ICC [2,3]. Nonresectable patients have been identified as having a poorer prognosis than patients undergoing surgery. The standard treatment for CCA is therefore surgery. The aim of the surgery is to achieve a radical resection while preserving an adequate liver function. Surgical resection is the only potentially curative option for CCA as survival after resection ranges between 25 and 40% at 5 years [2,3]. Unfortunately, due to the hidden and nonspecific symptoms of ICC in the early stages, most patients present at an advanced stage at diagnosis time. Nowadays, systemic therapy is the standard treatment for metastatic ICC patients with palliative intent and might improve the survival of these patients to a certain extent. According to the ABC-02 trial published by Valle et al. in 2010 [4], the combination of gemcitabine and cisplatin compared with gemcitabine alone reached a median overall survival (OS) of 11.7 and 8.1 months, respectively; and median progression-free survival (PFS) was 8.0 and 5.0 months, respectively. Gemcitabine and cisplatin became the standard of care in first-line settings, for both ICC and ECC patients. Only recently, the TOPAZ-1 phase III trial demonstrated positive results with the addition of an immune checkpoint inhibitor durvalumab to gemcitabine and cisplatin in an unselected CCA population. This combination conferred a 20% reduction in the risk of death compared with cisplatin and gemcitabine alone [5]. The landscape of therapeutic options has been rapidly evolving over the past decade with the possibility of new targeted therapies.

With the progress of the disease process, advanced ICC often preferentially metastasizes to the liver. From extrahepatic organs, the lungs, peritoneum and lymph nodes are the most common sites of metastases, though bones and brain metastases also occur. Patients with different metastatic sites might present different tumor biologic patterns and face different prognostic prospects and might need distinct therapeutic approaches. The incidence of bone metastases in ICC is around 14% according to different authors [6,7,8]. The role of the metastatic site on survival in CCA has already been investigated by some authors [6,7,8,9,10]. According to these studies, CCA patients with bone metastases have a particularly dismal prognosis. Yan et al. stratified ICC patients according to the metastatic site; respective median overall survival (OS) was 6 months for patients with liver metastases, 6 months for patients with lung metastases and 4 months for patients with bone metastases (*p* = 0.011). The authors demonstrated that patients with liver metastases have a better outcome compared with patients with bone metastases (for OS: liver vs. bone metastases: *p* = 0.003; liver vs. lung metastases: *p* = 0.112; bone vs. lung metastases, *p* = 0.130) [10].

Considering this poor prognosis, as well as the marginal survival benefit of palliative first-line treatment, we focused on ICC patients with bone metastases in this study. The aim was to retrospectively analyze the incidence of bone metastases in ICC patients in our series, and, to analyze their outcome in the terms of OS, PFS during first-line therapy and TTD (time from bone metastases occurrence to cancer death) to explore the importance of active anticancer treatment in this subgroup of ICC patients.

## 2. Materials and Methods

### 2.1. Patients and Follow-Up

A total of 186 metastatic ICC patients from three hospitals treated between January 2012 and December 2018 were retrospectively reviewed using electronic medical records. Only patients aged 18 years or more with a histologically confirmed diagnosis of intrahepatic cholangiocarcinoma were included, and all ICC patients with liver involvement only were non-amenable to surgery or any local therapy. Patients with a diagnosis of concomitant malignancy or a diagnosis different from adenocarcinoma were excluded. We collected basal clinical and pathological characteristics of our patients: age, sex, ECOG PS, histology, presence and site of metastases, first and second-line treatment and date of death. All patients underwent CT scan evaluation at the diagnosis and at disease evaluation every 3 months. CT-PET, MRI or bone scintigraphy was performed only in the presence of suspicious clinical symptoms or signs on CT imaging. The study protocol was approved by the Ethics Committee (protocol 78/2017/O/OSSN) which was in accordance with the Declaration of Helsinki and good clinical practice.

### 2.2. Statistical Analysis

Metastatic ICC patients were divided into three groups: Group A: with liver metastases only; Group B: all ICC metastatic patients other than Group A and C; Group C: with bone metastases (with or without other metastatic sites). Clinicopathologic and long-term survival data were collected and reviewed. Descriptive data were reported as mean with standard deviation (SD) in case of normal distribution and median and range if otherwise. The categorical variables were reported as absolute numbers and percentages. Overall survival (OS) was calculated from the date of metastatic ICC diagnosis to the date of death. The OS was censored at the last date of follow-up. Progression-free survival *(PFS*) was defined as the time of therapy initiation to disease progression or death. Time to death (TTD) was calculated from the end of first-line chemotherapy to patients’ death. The TTD was censored at the last date of follow-up. OS, PFS and TTD curves were constructed using the Kaplan–Meier method, and differences were analyzed using log-rank (Mantel–Cox) test. To outline the clinically relevant factors associated with predicting the impact of OS, we performed univariate and multivariate COX regression models to determine the joint association of several clinical factors investigated (ECOG PS, presence of bone metastases, second-line chemotherapy). The *p* value was bilaterally tested, and values less than 0.05 were regarded as statistically significant.

## 3. Results

### 3.1. Population Characteristics

Out of 186 metastatic ICC patients included in our study, 103 (55.4%) were male and 83 (44.6%) were female. The median age at diagnosis was 66.5 years (range 34–85 years). Dividing patients according to ECOG performance status, 100 (53.8%) ICC patients were ECOG PS 0, 51 (27.4%) ECOG PS 1, 28 (15.1%) ECOG PS 2 and 7 (3.8%) ECOG PS 3. Group A included 104 patients (55.9%), Group B 62 patients (33.3%) and Group C 20 patients (10.8%). A total of 12 patients (60%) out of 20 included in Group C and 8 patients (13%) out of 62 in Group B had three and more different metastatic sites. There was a difference between patients with good ECOG PS 0 and 1 and worse ECOG PS 2 and 3 favoring Group A and Group B. Group C with bone metastases had a higher proportion of patients with a poor performance status, though no statistically significant difference among the three groups was observed. Median OS in the whole group was 9 months (95% CI 7.4–10.6 months). Median PFS in the whole group was 4 months (95% CI 3.4–4.6 months). Moreover, 90 patients (48.4%) underwent at least two lines of anticancer treatments. The basic clinical–pathological characteristics of the 186 patients are detailed in Table 1. As shown in Table 1, no statistically significant difference was seen among the three groups for sex, age, ECOG PS and first-line gemcitabine-based chemotherapy.

### 3.2. Overall Survival in Metastatic ICC Patients

**Figure 1:**
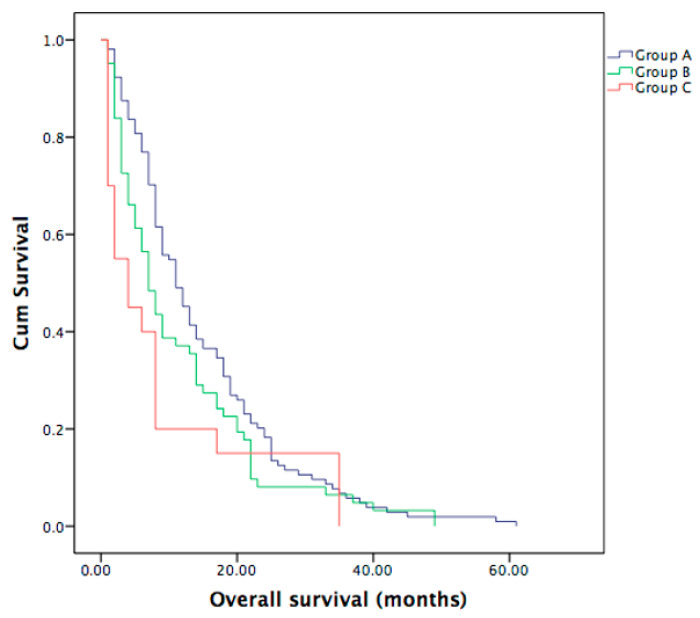
OS in Group C, in comparison to Group B (*p* = 0.234) and in comparison to Group A (*p* = 0.03). Median OS in Group A was 11 months (95% CI 8.3–13.6 months), in Group B was 7 months (95% CI 5.1–8.9 months) and in Group C was 4 months (95% CI 1.0–8.3 months).

Median OS was significantly different between the ICC patients with bone metastases (*n* = 20) and ICC patients with liver involvement only (*n* = 104), as shown in Figure 1. Median OS for the whole group was 9 months. Group C showed a shorter median OS of 4 months (range 1–35 months) in comparison to Group B (*n* = 62) with a median OS of 7 months (range 1–49 months), though not statistically significant (*p* = 0.234), and in comparison to Group A with a median OS of 11 months (range 1–104 months), with a statistically significant difference (*p* = 0.03). No statistically significant difference was seen between the Group A and B (*p* = 0.08), see also Figure 1.

### 3.3. Progression-Free Survival in Metastatic ICC Patients

All patients received gemcitabine-based chemotherapy in the first-line treatment. In general, the combination treatment (gemcitabine and cisplatin or oxaliplatin) was reserved for patients in good clinical condition (ECOG PS 0-1) without contraindications. The patients with poor clinical conditions (ECOG PS 2 or 3) or with contraindications were treated with gemcitabine in monotherapy.

**Figure 2:**
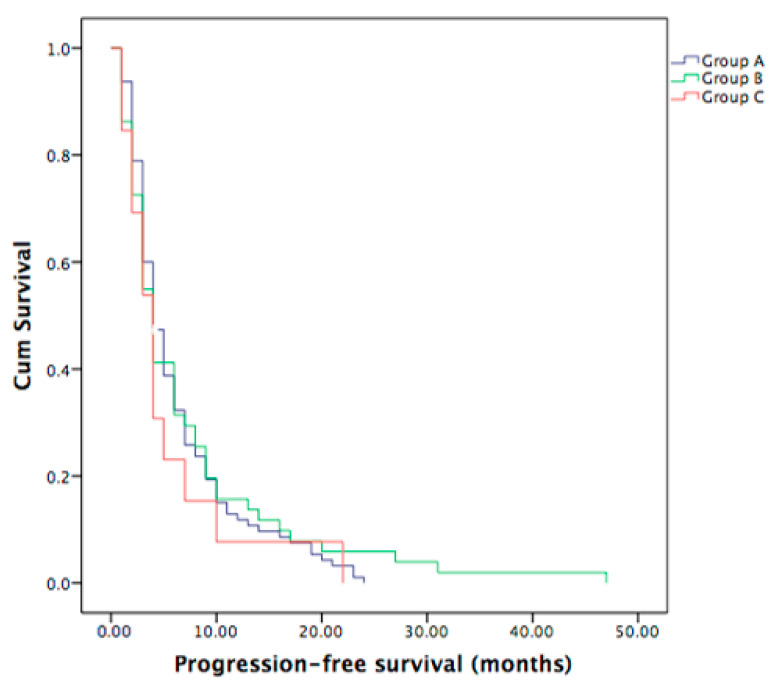
PFS did not differ between the three groups of patients (Group A vs. Group C with *p* = 0.44, Group B vs. Group C with *p* = 0.47, Group A vs. Group B with *p* = 0.68). Median PFS for all three groups was 4 months (95% CI 3.4–4.6 months).

Out of 159 metastatic PDAC patients who underwent the first-line chemotherapy, 95 were in Group A, 13 in Group B and the remaining 51 belonged to Group C. Median PFS for the whole group was 4 months, not statistically significant between the three groups (*p* = 0.813), as shown in Figure 2.

### 3.4. Second-Line Treatment in Metastatic ICC Patients

**Figure 3:**
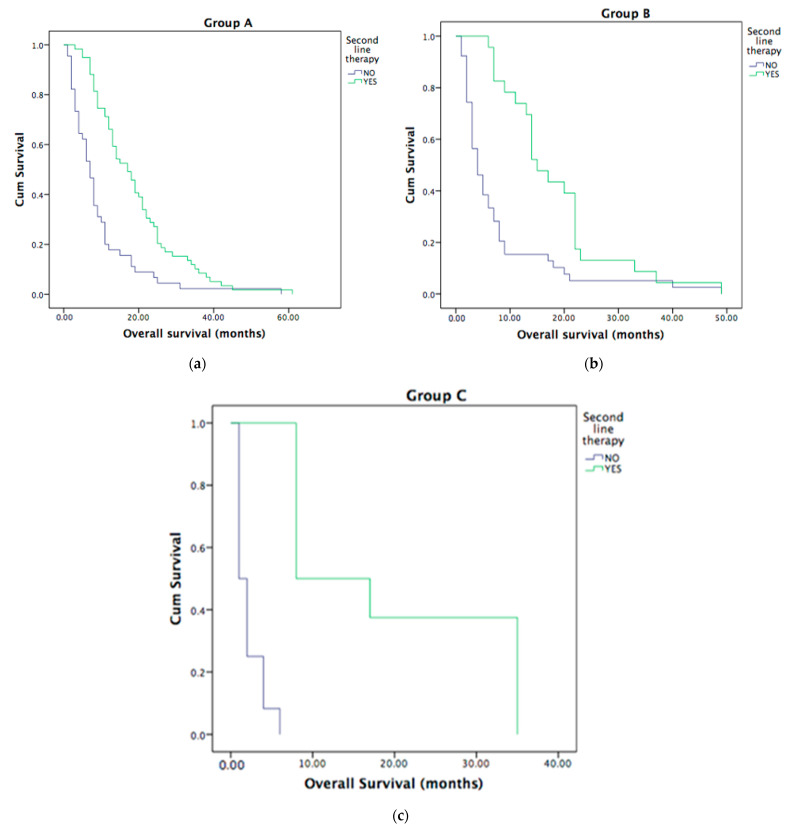
Second-line treatment and OS: better OS for ICC patients who underwent second-line treatment in Group A, *p* < 0.001 (**a**), Group B, *p* < 0.001 (**b**) and Group C, *p* < 0.001 (**c**).

The second-line treatment was administered to 90 (48.4%) of metastatic ICC patients. All three groups who underwent second-line treatment had better OS, as shown in Figure 3A–C. The median OS was 4 months (95% CI 1–8.3 months) in all groups.

### 3.5. Time from the End of First-Line Therapy to Death in Metastatic ICC Patients

**Figure 4:**
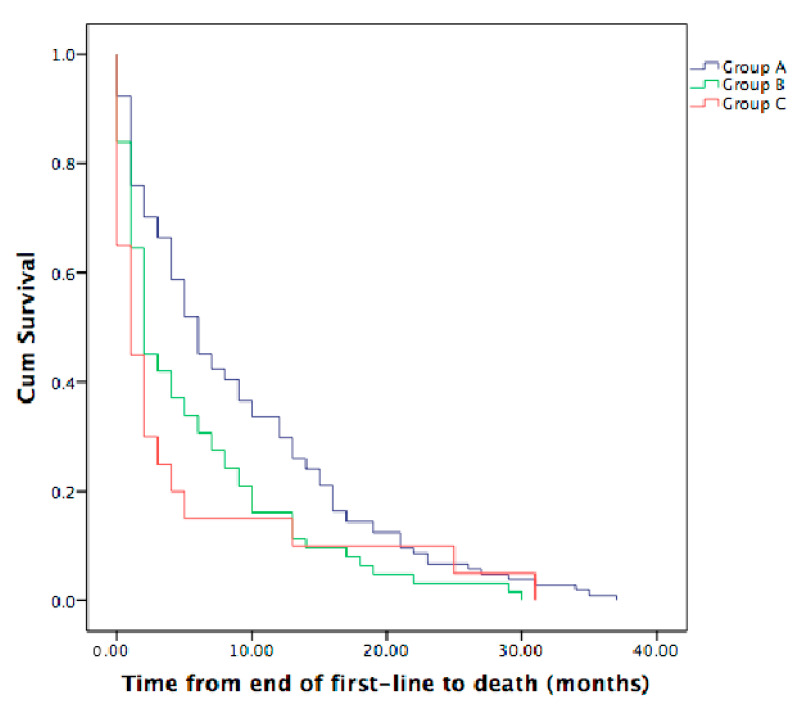
TTD with statistically significant difference between Group A and Group C (*p* = 0.016) and Group A and Group B (*p* = 0.007). Median TTD in Group A was 6 months (95% CI 4.2–7.8 months), in Group B was 2 months (95% CI 1.0–3.1 months) and in Group C was 1 months (95% CI 1.0–2.1 months).

The median time from the end of first-line therapy to death (TTD) was less than 1 month (range 1–31 months) for Group C, 2 months for Group B (range 1–30 months) and 6 months for Group A (range 1–37 months). We observed a statistically significant difference between Group A and Group C (*p* = 0.016) and also between Group A and Group B (*p* = 0.007), but not between Group B and Group C (*p* = 0.58), Figure 4.

### 3.6. ECOG PS of Metastatic ICC Patients

**Figure 5:**
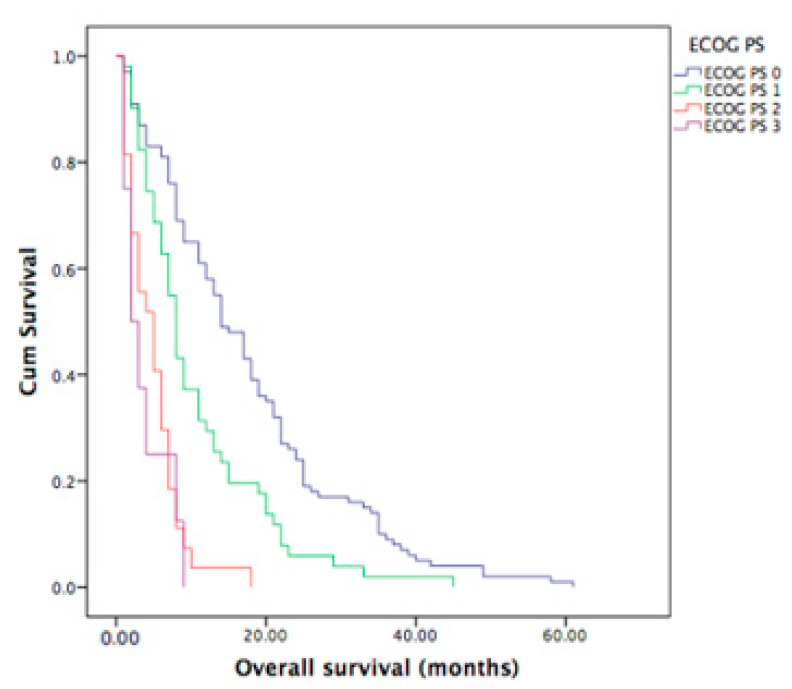
OS according to ECOG PS before first-line therapy initiation. The median OS for ECOG PS 0 group was 14 months (95% CI 10.7–17.3 months), in comparison to ECOG PS 3 with median OS of 2 months (95% CI 1.0–3.8 months).

There was a statistically significant difference in OS according to ECOG PS collected before initiation of the first-line treatment (*p* < 0.001). The median OS for ECOG PS 0, 1, 2 and 3 was 14, 8, 5 and 2 months, respectively (Figure 5).

In Group A (*n* = 104), 61 patients (58.7%) were ECOG PS 0, 29 (27.9%) were ECOG PS 1, 10 (9.6%) were ECOG PS 2 and 4 (3.8%) were ECOG PS 3, with a statistically significant difference (*p* = 0.00). In Group B (*n* = 62), 28 patients (45.2%) were ECOG PS 0, 20 patients (32.3%) were ECOG PS 1, 12 patients (19.3%) were ECOG PS 2, 2 patients (3.2%) were ECOG PS 3, with a statistically significant difference (*p* = 0.00). In Group C (*n* = 20), 11 patients (55%) were ECOG PS 0, 2 patients (10%) were ECOG PS 1, 6 patients (30%) were ECOG PS 2, 1 patient (5%) was ECOG PS 3, with a statistically significant difference (*p* = 0.04).

**Figure 6:**
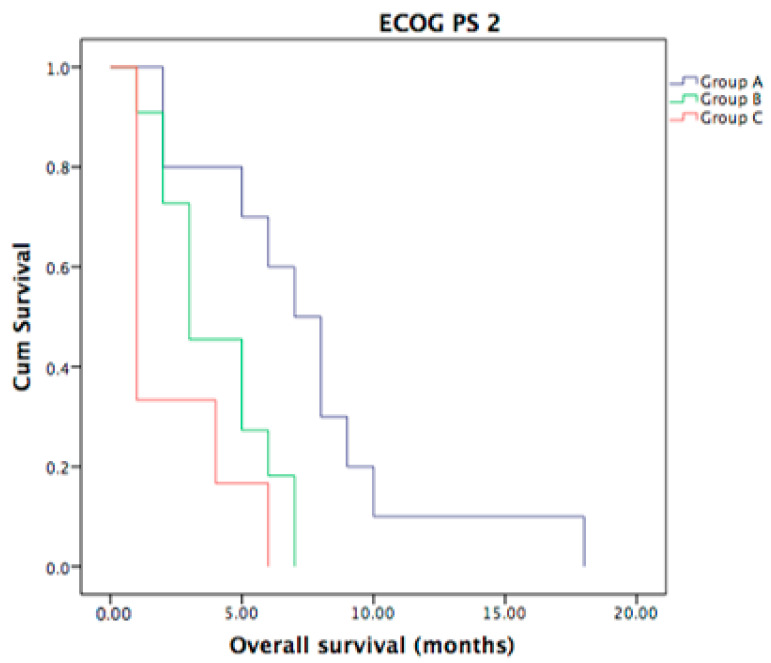
OS for ECOG PS 2 patients, with a statistically significant difference in OS between Group A and Group C (*p* = 0.003) and between Group A and B (*p* = 0.04).

Moreover, no statistically significant difference in OS was seen for the three groups (A, B, C) and ECOG PS 0 (*p* = 0.62) and ECOG PS 1 patients’ status (*p* = 0.18). However in ECOG PS 2 group, a statistically significant difference in OS was seen between Group A and Group C (*p* = 0.003) and between Group A and B (*p* = 0.04), but not between Group B and Group C (*p* = 0.07) as shown in Figure 6.

### 3.7. Univariate and Multivariate Prognostic Analyses

To outline the clinically relevant factors associated with predicting the impact of OS, we used univariate and multivariate COX regression models (Table 2 and Table 3). Univariate analysis revealed that ECOG PS 1 or higher, the absence of second-line treatment and the presence of bone metastases, were associated with a significantly higher HR for death (HR = 2.3; 95% CI = 1.7–3.2; *p* < 0.001, HR = 2.6; 95% CI = 1.9–3.5; *p* < 0.001 and HR = 1.6; 95% CI = 1.04–2.7; *p* = 0.033) (Table 2). We added all these variables to the multivariate analysis. A Cox proportional hazards model analysis was performed to determine the joint association of these clinically relevant factors (ECOG PS, second-line chemotherapy and patients’ Group A, B or C). The Cox proportional hazards model analysis showed that poorer ECOG PS, absence of second-line treatment and the presence of bone metastases were associated with a significantly higher HR for death (HR = 2.2; 95% CI = 1.6–3.1; *p* < 0.001, HR = 2.5; 95% CI = 1.8–3.4; *p* < 0.001 and HR = 2.2; 95% CI = 1.3–3.7; *p* = 0.01, respectively), see Table 3.

## 4. Discussion

Our study demonstrates that around 11% of ICC patients developed bone metastases in our series. This subgroup of ICC patients showed no differences in PFS to first-line chemotherapy but had a shorter median OS of 4 months compared to ICC patients with liver involvement only. From the end of first-line treatment, the residual OS was around 1 month in our study. Interestingly, this subgroup presented a poorer performance status and if treated ECOG PS 2 ICC patients with bone metastases, their outcome was worse in comparison to ICC patients with liver involvement only and poor performance status. Finally, the presence of bone metastases, poor performance status and no subsequent second-line treatment was associated with a worse outcome in multivariate analysis. This study makes us reflect on the usefulness of active first-line chemotherapy over best supportive care (BSC) in the subgroup of metastatic ICC patients with bone metastases, especially with poor performance status.

Cholangiocarcinomas are a heterogeneous group of neoplasms of the bile ducts [11,12] and represent the second most common hepatic cancer after hepatocellular cancer. Intrahepatic cholangiocarcinoma represents a malignant entity parting from epithelium cells of the intrahepatic bile ducts [13]. Defined by the anatomic localization, ICCs are distinguished from ECC (perihilar and distal cholangiocarcinoma) and gallbladder carcinoma. ICC and ECC show differences in risk factors, histopathologic features and prognosis. In Europe, this tumor frequently presents as a sporadic cancer in patients without defined risk factors and is usually diagnosed at an advanced stage with a consequent poor prognosis [14,15,16,17,18]. Cholangiocarcinomas commonly metastasize via the lymphatic system into regional lymph nodes and hematogenous metastasis to the liver, lungs and peritoneum. Bone metastases are common and might cause severe morbidity and mortality [19]. They usually occur in the axial skeleton. The common patterns of destruction are osteolytic or mixed osteolytic and osteosclerotic lesions [19]. For patients with unresectable advanced disease, the median OS is less than one year and poor performance status (ECOG ≥ 2) is the strongest prognostic factor [20]. Yan et al. [10] analyzed a total of 981 patients diagnosed with stage IV ICC. Of this population, 58% patients were diagnosed with liver metastases only, and 30% patients were diagnosed with bone metastases which is a higher proportion in comparison to our study. The median OS was 6 months for the liver metastases only group and 4 months for ICC patients with bone metastases, similar to our study. The authors performed an intergroup analysis which showed that patients with liver metastases have a better outcome compared with patients with bone metastases, as in our study. Han et al. [6] conducted a retrospective study investigating 186 metastatic ICC patients (from a total of 370 ICC patients, both metastatic and non-metastatic). The most common metastatic sites included bone metastases, detected in 14% of all patients. Interestingly, after detection of bone metastases, the residual OS was 4.4. months which was worse than the presence of peritoneal metastases. Interestingly, in multivariate hazard regression analysis, one of the predictors of poor survival was the absence of subsequent treatment. In accordance with authors of this study, regarding second-line treatments, this factor is influenced by a multiple factors such as performance status, liver function and previous treatments, therefore, it is not surprising that patients who received second-line treatment showed a better outcome in comparison to the BSC arm. Han et al. [6] concluded that the presence of distant metastases was associated with poor patient outcomes, though there was no significant difference between metastatic sites. On the contrary, Cheng et al. [7] classified 1567 metastatic ICC patients according to the metastatic sites. Compared with those with multiple-site metastases, patients with single-site metastases had better prognostic outcomes. In accordance to our study, liver metastases had better prognostic outcomes than bone metastases and the authors conclude that different metastatic sites have distinct impact on the survival outcomes of patients with advanced ICC [7]. The incidence rate of bone metastases in this study was 14.5%, with 4.3% of patients with bone metastases as single site metastases. The ENSCCA Registry [8], a multicenter observational study, included more than 2.000 CCA patients, both ICC and ECC, with 28.4% of metastatic CCA. Bone metastases were present in 13.8% of ICC patients. The mOS was 10.6 months for CCA patients receiving active palliative therapies and 4 months for those receiving BSC. Moreover, ECOG performance status was one of the independent prognostic factors of OS. In particular, in the whole CCA group, patients with ECOG PS 3 and 4 had a median OS of only 3 months [8].

This study has some limitations and bias. The main limitation is the retrospective character of our study; therefore, the study cannot drive any definitive clinical conclusions. Moreover, the number of the patients was moderate and all our patients were treated without durvalumab which showed an increase outcome in the TOPAZ-1 study [5]. Finally, the lack of a preplanned program of bone metastases’ diagnoses (all patients underwent CT scan evaluation, CT-PET only in the presence of suspicious clinical symptoms or signs on CT imaging) might have underestimated the real proportion of ICC patients with bone metastases.

From the clinical point of view, we can conclude that a good ECOG performance status might be a selection factor for active anticancer therapy versus best supportive care in metastatic ICC patients. To the best of our knowledge, this is the first study which evaluates both the prognostic and predictive significance of the bone metastatic site in ICC patients. As the residual OS after diagnosis of bone metastases in ICC patients is extremely poor, discussion with the patient, where possible, to share the decision about best supportive care might be acceptable, in order to avoid futile collateral effects of the palliative treatments and to maintain the quality of life of our patients for as long as possible.

## 5. Conclusions

The role of the metastatic bone site in ICC patients on survival and further management for metastatic ICC remains to be explored. Further studies are needed to investigate how much this subgroup of ICC patients with bone metastases really benefits from active treatment, in comparison to best supportive care. More prognostic biomarkers are needed to answer this question, however, a poor ECOG performance status before treatment initiation might be an indication for best supportive care and not active treatment, the decision about which has to be shared with our patients.

## Figures and Tables

**Table 1:** Clinical characteristics of the three groups (Group A, Group B and Group C).

	Group A (Patients with Liver Metastases Only)	Group B (Patients Other than Group A/C)	Group C (Patients with Bone Metastases)	*p*
Number of patients	104	62	20	
Sex				
Male	56 (54%)	36 (58%)	11 (55%)	
Female	48 (46%)	26 (42%)	9 (45%)	NS (*p* = 0.87)
Age				
more than median age	46 (44%)	34 (55%)	12 (60%)	
less than median age	58 (56%)	28 (45%)	8 (40%)	NS (*p* = 0.18)
ECOG PS				
0	61 (59%)	28 (45%)	11 (55%)	
1	29 (28%)	20 (32%)	2 (10%)	
2	10 (9%)	11 (18%)	6 (30%)	
3	4 (4%)	3 (5%)	1 (5%)	NS (*p* = 0.14)
First-line Gemcitabine-based chemotherapy	104 (100%)	62 (100%)	20 (100%)	

NS = not statistically significant.

**Table 2:** Univariate Cox analysis of overall survival.

Variable	HR	95.0% CI for HR		*p*
		Lower	Upper	
ECOG PS	2.377	1.752	3.226	<0.001
Second line therapy	2.613	1.939	3.523	<0.001
Groups				
Group B	1.286	0.937	1.765	0.120
Group C	1.693	1.043	2.748	0.033

**Table 3:** Multivariate Cox analysis of overall survival.

Variable	HR	95.0% CI for HR		*p*
		Lower	Upper	
ECOG PS	2.258	1.637	3.113	<0.001
Second line therapy	2.526	1.843	3.463	<0.001
Groups				
Group B	1.151	0.835	1.587	0.391
Group C	2.296	1.399	3.769	0.001

## Data Availability

The data presented in this study are available on request from the corresponding author.
